# Effectiveness of mindfulness training and dietary regime on weight loss in obese people

**Published:** 2015

**Authors:** T Asadollahi, S Khakpour, F Ahmadi, L Seyedeh, Tahami, S Matoo, H Bermas

**Affiliations:** * Department of Psychology, Karaj Branch, Islamic Azad University, Karaj, Iran; ** Department of Psychology, Sari Branch, Islamic Azad University, Sari, Iran; *** Department of Management, Tehran Branch, Payame Noor University, Tehran, Iran; **** Department of Psychology, Asalouyeh Branch, Payam Noor International University of Asalouyeh, Asalouyeh, Iran

**Keywords:** mindfulness training based on cognitive therapy, diet, obesity

## Abstract

The present research was aimed to investigate the effectiveness of mindfulness training and dietary regime on weight loss in obese people. The research was quasi-experimental with posttest-pretest that used control group. The population consisted of all the individuals who attended two clinics of nutrition advice and diet therapy in Karaj. 60 individuals, whose BMI was more than 30, were selected by using the random sampling method. Moreover, they were evaluated by using the SCL-90 test in order to neglect them in case there existed any other significant disorder. Next, they were selected based on age, sex, and education. After explaining the individuals the ongoing research and collecting the informed consent written by them, the samples were placed in four groups (15 in each group). The groups that received mindfulness training attended the nutrition center for eight to 120-minute sessions. In addition, since all the participants referred to the center were motivated to lose weight, individuals who were placed in the control group and those who received mindfulness training were asked not to follow any specific diet for two months. Moreover, the in depth relaxation CD was prepared for those who asked, in order to train themselves at home. Descriptive statistical methods were employed in order to analyze the data and ANACOVA and variance analysis with frequent measurement were used. The research findings indicated that mindfulness training was accompanied by diet, which resulted in weight loss in obese patients. In addition, the findings of the two-month follow-up indicated lasting results.

## Problem statement

In the recent years, changes in lifestyle led the people to face with a new dimension of nutrition disorder, meaning overweight and obesity, so that this has become a new problem in public health [**1**]. Fat metabolism disorder often shows itself as a chronic disease named obesity. Obesity is the most important public health challenge in the present era and the health sector in most countries involves in the rising incidence of obesity and its complications [**2**]. According to a report published by the World Health Organization (WHO) in 2008, more than 1.4 billion people in the world deal with overweight, 200 million of them are male and 300 million are female. It was predicted that 1.3 billion of people in the world will be overweight and 53 billion will be obese in 2020. Obesity is not caused only by physiological factors but it is also influenced by the interplay of psychological and physiological factors, and has been classified as a disease by the medical and health centers [**3**]. Undoubtedly, a high percentage of different age groups deal with obesity in Iran [**4**]. It is not clear if obesity is an independent risk factor for public health or it can endanger people’s health by preparing the ground for the development of some diseases and cancers. Related researches indicated that obesity is accompanied by psychical effects such as polycystic ovary syndrome, type 2 diabetes, blood pressure, lipid disorders, cardiovascular diseases, asthma, cancer, arthritis, gout, skin disorders, liver disease, and shortness of breath in sleep. In addition, it has psychological effects such as depression, reduction in self-confidence, increase in anxiety and aggression [**5**]. Some of the studies were also conducted with the aim of investigating the relationship between obesity and the psychological well-being [**6**]. Moreover, the indirect expenses of obesity such as the reduction in capability, increase in illness period, premature exhaustion, insomnia caused by depression and osteoarthritis pains in the joints that bear the weight and social consequences such as injustice. Although the psychological factors are of fundamental importance in the development of obesity, obviously, it is not clear to what extent these factors result in death [**3**]. There was a positive correlation between obesity and depression in many researches [**7**]. Lamertz believed that obesity leads to the creation of a negative attitude, psychological stress, and increase in psychological disorders [**8**]. There is a variety of methods to reduce obesity, among which diet could be named in the meantime. There are many evidences that prove failure of dietary regimes including commercial aspects, body’s ability to adapt to difficult situations and a wide range of neurotransmitters in the phenomenon of weight control. In addition, relevant studies demonstrated that one-third to two thirds of the individuals who use only dietary regimes to reduce their weight, could not continue the procedure successfully [**9**]. The imperfect cycle of dietary regimes is repeated due to the failure in weight loss and leads to depression, severe mood swings, insomnia, sadness, tears, and other symptoms [**5**]. Accordingly, the strength of cognitive infrastructures in the etiology of obesity could be mentioned [**10**]. However, according to Johnson, the cognitive approach is not developed in explaining obesity, especially our understanding of specific processes which are involved in the emergence and maintenance of cognitive symptoms that are effective in eating is limited [**10**]. Since it is associated with a complex causal network, obesity is associated with a complex causal network, interventions to improve it are also complex and the demand for a combination of several approaches, thereby combined methods are more effective in the reduction of obesity. Recent developments in the treatment of psychological disorders and the reduction of obesity led to the development of novel and new methods by the clinical psychologists [**11**]. In this regard, interventions based on mindfulness, which is deemed as a third generation behavioral-cognitive therapy and considered as a form of meditation, originating in the Eastern religious teachings and rituals, are employed [**12**]. Recently, therapists have deduced that mindfulness has a great impact on the elaboration of orderly and restrained eating patterns [**13**]. Kabat-Zinn defined mindfulness as paying attention to purposeful, specific, and without judgment and prejudice methods [**14**]. Marshal [**37**] emphasized the necessity and importance of the inclusion of mindfulness as one of the major components of psychological treatments. Hence, there have been many interventions and treatments based on mindfulness [**15**]. The preliminary empirical evidences indicated that mindfulness could be involved in obesity. For instance, Dalen et al. (2010) indicated in a research entitled “mindful Eating and Living (MEAL): Weight, eating behavior, and psychological outcomes associated with a mindfulness-based intervention for people with obesity” that conscious eating has an impact on weight changes and dietary habits of obese people. Kristeller and Hallet (1999) referred to the effects of the cognitive therapy method based on mindfulness on eating disorder. It seems that “paying attention” is the main mechanism of mindfulness, because the frequent concentration and focus on a neutral stimulus such as breathing creates a proper attention atmosphere [**16**]. When a person is informed about his current status, he/ she does not pay attention to future [**17**-**19**]. Therefore, conscious eating helps people be informed of their physical and emotional desires thoroughly and changes their mental moods in any interaction with food and eating. It can be said that using mindfulness practices associated with dietary regimes prepared for the person particularly is a novel work that is expected to lead to weight loss. Therefore, the present research aimed to present a comprehensive training package based on Mindfulness-based Cognitive Therapy (MBCT) together with a proper and practical dietary regime.

## Methodology

The research was quasi-experimental with posttest-pretest that used control group. The population consisted of all the individuals who attended two clinics of nutrition advice and diet therapy in Karaj. Next, 60 individuals whose BMI was of more than 30 were selected by using the random sampling method. Moreover, they were evaluated by using SCL-90 test in order to neglect them in case there existed any other significant disorder. Afterwards, they were selected based on age, sex, and education. After explaining the individuals the ongoing research and collecting the informed consent written by them, the samples were placed in four groups (15 in each group). It should be mentioned that the minimum sample population in the experimental studies is 15 individuals [**20**]. The first experimental group was a group that received only a dietary regime and the second one only received mindfulness training based on cognitive therapy. The third experimental group received a mindfulness training based on cognitive therapy together with a dietary regime. The control group received no treatment. It should be mentioned that individuals were weighed by a portable digital scale. Descriptive statistical methods were employed in order to analyze the data and ANACOVA and variance analysis with frequent measurement were used. The research instruments for the collection of data were the following: demographic characteristics questionnaires and revised list of psychiatric symptoms of SCL-90-r. 

**Demographic characteristics questionnaires**

This questionnaire was provided to collect regular personal information of participants including age, education, economic and social status, etc.

**Revised list of psychiatric symptoms of SCL-90-r**

A revised list of psychiatric symptoms of SCL-90-r was developed by Lipman and Covi in 1973 in order to show the psychological aspect of mental and physical patients [**21**]. Douketis et al. (1976) reviewed the mentioned questionnaire and prepared the final edition with the revised list of psychiatric symptoms entitled SCL90-r which consisted of 90 5-point questions (none = 0, a little = 1, somewhat = 2, high = 3, very high = 4). Moreover, it had nine subscales including somatization, obsession, interpersonal sensitivity, depression, anxiety, aggression, phobia, paranoid thoughts, and psychosis. Deragotis, Rickels and Rock (1676) calculated the concurrent criterion validity coefficients of nine dimensions of the test to be between 36% and 73%, of which all were significant at 1% level [**22**]. The domestic version of the reliability and validity of the questionnaire was investigated by Mirzaie R (1980) [**35**] and it was in line with the American version (Anisi et al., 2011). Furthermore, Mar'ashi (1996) found the reliability coefficients of the test to be between 80% and 94% by using Cronbach’s alpha method (Albukordi, 2012). In addition, after the implementation of the questionnaire in the present research, the reliability coefficients related to its dimensions were calculated to be 67% to 87% by using Cronbach’s alpha method.

**Body Mass Index**

The Body Mass Index is obtained through dividing weight (in kilograms) by height squared (in meters). Weight was measured by using a digital scale with a sensitivity of 100 g and height was measured by using a wall stadiometer with an accuracy of 5.0 cm (in a standing position and without shoes).

**Digital scale**

Prior to the intervention during the 8 sessions for reducing weight, the participants were weighed each time. Their weights were recorded in their file in kilograms. The participants in the control group were only weighed in the first and last sessions.

**MBCT training program**

The instruction of the sessions was based on the handbook of “Mindfulness-based Cognitive Therapy” written by Rebecca [**36**]. This intervention consisted of 8 training sessions which lasted for 2 hours once a week. In order to carry out the trainings, the relaxation in depth CD and book were used (Bahadoran & Pour Naseh, 2003). It should be said that as the authors mentioned in the beginning of their books, theories are basically based on cognitive sciences with references to the history of Buddhism.

Explanations: in this exercise, the interrelationships between the presence of mind and the training sessions showed how and to what extent our problems emerge and last. Weeks 6, 7, and 8 are in fact a program to prevent recurrence. In other words, lessons during these three weeks resulted in the realization of unique patterns of emotional response and identification of negative thoughts as warnings.

First session: the most important part of this session was the creation of a protective atmosphere in the field of the subject in the following week. The participants were introduced and explained their expectations afterwards. In summary, the following tips were mentioned in the first session:

1- Eating a raisin with awareness

2- Body checking meditation

3- Speaking about the experience gained in the meeting

4- Presenting a list of assignments which had to be carried out at home

5- Presenting a chart about unpleasant events in order to be completed that week

Second session: most of the participants had a challenge in this session. Focusing on the body with greater clarity showed the reality of mental reactions. Focusing on the feeling and thinking created a special atmosphere in which the person found out the way of interpretation of the situation which led to his feeling and not only the situation. In summary, the following tips were mentioned in the second session:

1- Body checking excises

2- Breathing for three minutes associated with mindfulness and mind meditation

3- Thinking about the exercises and feeling any one of them accurately and precisely

4- Reviewing and discussing about exercises carried out the week before

5- Presenting assignments in order to be done at home

6- Presenting a chart about unpleasant events in order to be completed in that week

Third session: After two training sessions of body checking, the participants were trained to consider their body as a place in which experiences could be realized. A thorough attention to breathing provided a new world that revealed how the process of breathing affected us. Through deep searching in their experiences, participants would be aware of many constructive components out of their experiences (physical feelings, thoughts and emotions) at any moment. Generally, the following tips were mentioned in the third session:

1- Smart movement

Relearning the way of paying attention to physical experiences at any moment

Awareness of body experience on the movement

Experience with the adoption of the present

2- Practicing informed listening 

3- Three minutes of breathing

4- Presenting pleasant experiences which had to be recorded during the last week and discussing them

5- Presenting home assignments

Fourth session: being in the present was trained in the fourth session. In summary, the following tips were mentioned in the fourth session:

1- Listening or watching for three minutes associated with mindfulness (awareness of the breath, body parts, sounds, thoughts and informed choices)

2- Three minutes of breathing

3- Walking with mindfulness

4- Discovering and defining depression, anxiety, self-esteem and psychological factors related to obesity

5- Presenting home assignments

Fifth session: in this session, it has been emphasized that affairs should be felt and experienced as they were, without any judgment and trying to show them differently. In fact, a new method of communication was trained. Participants were led to the recognition of typical patterns to meet their various experiences. In summary, the following tips were mentioned in the fifth session:

1- Meditation sessions

2- Emphasizing how to react to whatever we feel or think about and whatever results from physical feelings

3- Three minutes of breathing

4- Reading poems from Sohrab Sepehri and interpreting their contents

5- Presenting home assignments

Sixth session: participants were taught that thoughts are not facts for sure. In addition, ways to exit negative thoughts and moods were trained and it was thought that individuals could use them to study their experiences more accurate. This process of recognizing the existing patterns of thought could help us think thoroughly and seek for frequent answers. Moreover, it could help individuals achieve a broader approach about the process of our thinking in order to work on them with an approach based on the review, curiosity, and serenity. In summary, the following tips were mentioned in the sixth session:

1- Meditation session

2- Addressing the participants’ problems during practicing and discovering their effects on the body and mind

3- Three minutes of breathing

4- Being prepared for the end of the period

5- Raising personal recurrence and working on it in order to eliminate it

6- Presenting home assignments

Seventh session: In this session, participants were trained to take care of themselves in the best way. Their attention was drawn to the subject that merely reacted to current experience. They were taught to use any recognition and identification they acquired during the process and try to enjoy from the beginning of the process. In addition, they were aware of the personal patterns and vulnerability. They also learned to employ their own increasing awareness. In summary, the following tips were mentioned in the seventh session:

1- Three minutes of breathing and presenting problems and issues during practicing and finding their impact on mind and body

2- Identifying recurrences and activities that would cause a recurrence again

3- Walking consciously 

4- Body checking meditation

5- Presenting home assignments

Eighth session: how to use such cases in future decision making was trained in this session. Supporting participants about the possible positive changes might have emerged in them during the program and were emphasized in this session. In summary, the following tips were mentioned in the eighth session:

1- Body checking meditation, end of sessions

2- Study of the warning system and action plan presented for adoption at the time of recurrences and study of what was the most important thought in one’s life in each period in order to make the person capable of using it.

3- Creating some questions in the participants’ mind in order to answer to their own personal reflections during this program

4- Presenting some suggestions for the graduated students

**Research method**

The population consisted of all the individuals who attended two clinics of nutrition advice and diet therapy in Karaj. In this regard, some posters about training mindfulness by university postgraduate students were installed on the bulletin board. Afterwards, 260 participants were tested by using SCL-90-r test. Next, 60 individuals whose BMI was more than 30 were selected by using the random sampling method. It should be mentioned that individuals who took part in the present research were all under the supervision of the same dietitian in order to avoid affecting the research reliability. In addition, in order to evaluate the eating disorders and obesity at the same time, such as Bulimia Nervosa and Binge-Eating Disorder, dietitians were asked to not enter into the research obese people who simultaneously suffered from another eating disorder. As mentioned, SCL-90-r revised questionnaire of mental symptoms was filled by the research participants in order to evaluate the comorbidity of mental disorders and their impact.

After the referees were screened in terms of having the research criteria and explaining individuals the ongoing research and collecting the informed consent written by them, the samples were placed in four groups (15 in each group). The groups that received mindfulness training attended the nutrition center for eight to 120-minutes sessions. In addition, since all the participants referred to the center motivated to lose weight, individuals who were placed in the control group and those who received mindfulness training were asked not to pursue any specific diet for two months. Moreover, an in depth relaxation CD was prepared for those who asked in order to train themselves at home [**23**].

**Research findings**

**Table 1:** Descriptive statistics related to the age of participants in different groups

Group	Average	Mean	Variance	Standard deviation
Receiving dietary regime	44.7	46	146.92	12.121
Receiving mindfulness	45.60	48	169.25	13.01
Receiving a combination of mindfulness and dietary regime	43.80	44	183.88	13.56
Control	40.13	38	176.55	13.28

According to **Table 1**, it was specified that the average age of the participants who received dietary regime was 44.7 ± 12.121. In addition, the age of the participants who received mindfulness training was 45.60 ± 13.010. Moreover, the average age in the group that received a combination of dietary regime and mindfulness training was 43.80 ± 13.56 and this value was equal to 40.13 ± 13.28 in the control group.

**Table 2:** Descriptive statistics related to the height of participants in different groups

Group	Average	Mean	Variance	Standard deviation
Receiving dietary regime	166.66	167	62.59	7.91
Receiving mindfulness	169.80	169	198.67	14.09
Receiving a combination of mindfulness and dietary regime	165.03	165	110.94	10.53
Control	166.80	166	108.02	10.63

As it was obvious in **Table 2**, it was specified that the average height of the participants who received a dietary regime was 166.66 ± 7.91 and the average height of the participants who received mindfulness training was 169.80 ± 14.09. Moreover, the average height in the group that received a combination of dietary regime and mindfulness training was 165.03 ± 10.53 and this value was equal to 166.80 ± 10.63 in the control group.

**Table 3:** Scores of Body Mass Image (BMI) in the pretest

Variable->		BMI	
↓Group	Average	Variance	Standard deviation
Receiving dietary regime	166.66	62.59	7.91
Receiving mindfulness	169.80	198.67	14.09
Receiving a combination of mindfulness and dietary regime	165.03	110.94	10.53
Control	166.80	108.02	10.63

According to **Table 3**, it was obvious that the average BMI of the group that received dietary regime was 37.19 ± 5.24 in the pretest and the average of this variable was 34.31 ± 3.34 in the group that received mindfulness training. In addition, the average value of BMI in the group that received both dietary regime and mindfulness training was equal to 35.81 ± 4.87 and it was equal to 34.31 ± 3.07 in the control group. This finding indicated that the values of the average BMI were approximately equal to each other in the four groups and this showed that the groups were identically selected.

**Table 4:** Scores of the participants’ weight in the pretest

Variable->		Weight	
↓Group	Average	Variance	Standard deviation
Receiving dietary regime	101.46	392.29	19.80
Receiving mindfulness	91.08	199.80	14.13
Receiving a combination of mindfulness and dietary regime	106.87	483.81	21.99
Control	95.61	86.93	9.32

According to **Table 4**, it was shown that the average weight of the group that received dietary regime was 101.46 ± 19.80 and the average of this variable was 91.08 ± 14.13 in the group that received mindfulness training. In addition, the average weight in the group that received both dietary regime and mindfulness training was equal to 106.87 ± 21.99. This finding indicated that individuals who were in the group that received mindfulness training were heavier than others and those who received only dietary regime were lighter than others.

**Table 5:** Scores of Body Mass Image (BMI) in the posttest

Variable->		BMI	
↓Group	Average	Variance	Standard deviation
Receiving dietary regime	33.77	43.77	6.61
Receiving mindfulness	29.38	11.43	3.38
Receiving a combination of mindfulness and dietary regime	28.11	21.85	4.67
Control	34.35	8.87	2.97

According to **Table 5**, it was obvious that the average BMI of the group that received dietary regime was 33.77 ± 6.61 in the posttest and the average of this variable was 29.38 ± 3.38 in the group that received mindfulness training. In addition, the average value of BMI in the group that received both dietary regime and mindfulness training was equal to 28.11 ± 4.67 and it was equal to 34.35 ± 2.97 in the control group. This finding indicated that the values reduced compared to the pretest.

**Table 6:** Scores of participants’ weight in the posttest

Variable->		Weight	
↓Group	Average	Variance	Standard deviation
Receiving dietary regime	92.47	485.58	21.41
Receiving mindfulness	78.62	196.58	14.02
Receiving a combination of mindfulness and dietary regime	84.28	421.22	20.52
Control	95.77	85.81	9.26

As it was shown in **Table 6**, the average weight of the group that received dietary regime was 92.47 ± 21.41 in the posttest and the average of this variable was 78.62 ± 14.02 in the group that received mindfulness training. In addition, the average weight in the group that received both dietary regime and mindfulness training was equal to 84.28 ± 20.52 and it was equal to 95.77 ± 9.260 in the control group. This finding indicated that the values (except for the control group) reduced compared to the pretest.

**• Dietary regime leads to weight loss in obese people**

**Table 7:** ANACOVA of weight loss variable

Statistical indices ->	Sum of squares	Df	Mean squares	F	Sig.	Effect size
↓Sources of variance						
Impact of intervention	3913.092	1	2616.076	776.134	0.000	0.422
Error	1160.931	27	776.134			
Total	234273.740	30				

The significance level in **Table 7** was smaller than 0.05, therefore, it could be expressed that the variable of weight in individuals who received dietary regime in the posttest had a significant difference in the value in the pretest. In addition, a positive value of the effect size showed that this variable had a progress in the posttest. On the other hand, since the value of the effect size was equal to 0.422, it could be deduced that the dietary regime had about 43% impact on the weight loss of obese individuals. Hence, the progress in this field meant that the variable of weight in the experiment group reduced compared to the control group. Therefore, the research hypothesis was approved.

**• Mindfulness training leads to weight loss in obese people**

**Table 8:** ANACOVA of weight loss variable

Statistical indices ->	Sum of squares	Df	Mean squares	F	Sig.	Effect size
↓Sources of variance						
Impact of intervention	6924.999	1	6924.999	493.651	0.000	0.948
Error	3170.850	27	3170.850			
Total	251250.030	30				

The significance level in **Table 8** was smaller than 0.05, therefore, it could be expressed that the variable of weight in individuals who received mindfulness training based on cognitive therapy in the posttest had a significant difference in the value in the pretest. In addition, the positive value of the effect size showed that this variable had a progress in the posttest. On the other hand, since the value of the effect size was equal to 0.948, it could be concluded that mindfulness training based on cognitive therapy had about 95% impact on the weight loss of obese individuals. Hence, the progress in this field meant that the variable of weight in the experiment group reduced compared to the control group. Therefore, the research hypothesis was approved.

**• Mindfulness training associated with dietary regime leads to weight loss in obese people**

**Table 9:** ANACOVA of weight loss variable

Statistical indices ->	Sum of squares	Df	Mean squares	F	Sig.	Effect size
↓Sources of variance						
Impact of intervention	6786.374	1	6786.374	19.708	0.000	0.966
Error	609.643	27	609.643			
Total	273479.310	30				

The significance level in **Table 9** was smaller than 0.05, therefore, it could be expressed that the variable of weight in individuals who received mindfulness training based on cognitive therapy and appropriate dietary regime in the posttest had a significant difference in the value in the pretest. In addition, a positive value of the effect size showed that this variable had a progress in the posttest. On the other hand, since the value of the effect size was equal to 0.966, it could be concluded that mindfulness training based on cognitive therapy and appropriate dietary regime had about 97% impact on the weight loss of obese individuals. Hence, the progress in this field meant that mindfulness training based on cognitive therapy associated with appropriate dietary regime had a better impact on weight loss of obese people compared to the condition that they only received mindfulness training or dietary regime.

**• Mindfulness training based on cognitive therapy associated with appropriate dietary regime has a better effectiveness on weight loss of obese individuals compared to those who only receive mindfulness training.**

**Table 10:** ANACOVA of weight loss variable

Statistical indices ->	Effect size
↓Group	
Combination (mindfulness and dietary regime training)	0.966
Mindfulness training	0.948

As it was obvious in **Table 10**, the effect size related to the group only trained in mindfulness (0.948) was less than the group trained in mindfulness and dietary regime (0.966). Therefore, it could be concluded that our hypothesis was approved; thereby mindfulness training had less impact compared to the training of mindfulness and dietary regime together.

**• Effectiveness of dietary regime on weight loss of obese individuals has durability after two months.**

**Table 11:** Descriptive statistic of weight loss variable

Group	Criterion->	Average	Standard deviation	Number
	↓Variable			
Individuals who received dietary regime	Weight in posttest	92.47	21.41	15
	Weight after two months	99.23	21.44	15

**Table 12:** Tests related to the impact of dietary regime after two months

Group	Tests	Value	Observed F	Df	Sig.	Effect size
Individuals who received dietary regime	Pillai's Trace	0.495	13.725	14	0.002	0.495
	Wilks' Lambda	0.505	13.725	14	0.002	0.495
	Hotelling's Trace	0.980	13.725	14	0.002	0.495
	Roy's Largest Root	0.980	13.725	14	0.002	0.495

Based on **Table 12**, since the value of P was greater than 0.05, it could be concluded that the weight of the individuals had a significant difference after two months. According to the descriptive statistics and referring to the average weight of individuals, the weight of participants increased after two months and the dietary regime alone could not have durability.

**• Effectiveness of mindfulness training and dietary regime on weight loss of obese individuals has durability after two months.**

**Table 13:** Descriptive statistic of weight loss variable

Group	Criterion->	Average	Standard deviation	Number
	↓Variable			
Individuals who received dietary regime and were trained in mindfulness	Weight in posttest	84.2867	20.52364	15
	Weight after two months	84.1467	20.55859	15

**Table 14:** Tests related to the impact of mindfulness and dietary regime after two months

Group	Tests	Value	Observed F	Df	Sig.	Effect size
Individuals who received dietary regime and were trained in mindfulness	Pillai's Trace	0.074	1.114	14	0.309	0.074
	Wilks' Lambda	0.926	1.114	14	0.309	0.074
	Hotelling's Trace	0.080	1.114	14	0.309	0.074
	Roy's Largest Root	0.080	1.114	14	0.309	0.074

Based on **Table 14**, since the value of P was greater than 0.05, it could be concluded that the weight of the individuals had no significant difference after two months. Mindfulness training based on cognitive therapy and dietary regime had durability in the individuals’ weight loss.

## Conclusion

The present research indicated that mindfulness training based on cognitive therapy had an impact on the control of obesity. Although there were a limited number of studies that directly investigated the impact of mindfulness training based on cognitive therapy on the reduction of obesity, the present research demonstrated this effectiveness. Mindfulness training is a method in which the patient is informed about the origins of the disorder and its mechanism in the brain. Moreover, this method prevents the patient from being nervous, concentrates on his thoughts and tendencies in the state of consciousness, makes the person capable of not choosing repeating actions or thoughts and ruminating them to reduce anxiety and helps him think about the biological origins of the disorder. Although human technology, growth, and progress allow him to eliminate physical movement in the treatment of many diseases and save time and money, it should be expressed that many of the diseases and mental disorders could be treated by using short-term trainings. Patients who do not have an adequate literacy cannot be trained by using non-attendance treatment methods and what some of the researchers have mentioned about the treatment of psychological disorders by no direct presence and interaction between the references and the therapists, demands further investigations. Generally, the mindfulness method has a great impact on obese individuals because mindfulness training leads to cognitive changes in the way of thinking and actions of patients and takes advantage of conditional strengthening principals. Therefore, the patient makes an effort to reach to the next step and see himself above and this tendency to a continuous improvement leads to better conditions gradually. At the same time, he continues his personal treatment in peace and awareness and resolves his deficiencies in person meetings.

First hypothesis: dietary regime leads to weight loss in obese individuals.

According to the findings of the research, it was specified that dietary regime alone had an impact on obesity of people and their weight presented a considerable reduction. Moreover, the average value of weight of the individuals in the experiment group was reduced after the implementation of the dietary regime compared to the average weight in the control group. The significance level was equal to 0.000, which was less than 0.05. In addition, the impact of the dietary regime was 43% (43% of the variance was explained by the test implementation). Since obesity is one of the most threatening issues and of the most significant non-communicable diseases (WHO, 1998), the necessity to correct the pattern of food consumption seems to be required. This is applicable through nutrition advice and proper and continuous education and training. The effectiveness of the weight loss regime demands frequent references and sufficient incentives of the referees. An appropriate regime needs to be given according to the weight and BMI of referees and changes in BMI and weight should be measured in the next sessions in order to perform the necessary changes in addition to calculate its success and they must be told to the person. The results of the present research indicated that the low-calorie diet had an impact on weight loss of individuals with obesity, which was in line with the findings of relevant studies such as the ones of Navidian A, Abedi MR, Baghban I, Fatehizadeh M, Poursharifi H, Hashemi Dehkordi M (2010), Mazloom Z, Kazemy F, Tabatabaie SH, Ansar H (2009), West DS, Gorin AA, Subak LL, Foster G, Bragg C, Hecht J et al (2011), Foster GD, Wyatt HR, Hill JO, Makris AP, Rosenbaum DL, Brill C et al (2010) [**24**-**27**].

Second hypothesis: mindfulness training leads to weight loss in obese individuals.

According to the findings of the present investigation, it was specified that mindfulness training had a considerable impact on weight loss among individuals with obesity since the average weight of the participants in the experiment group was reduced after they received mindfulness training based on cognitive therapy compared to those who received the training in the control group. The significance level was equal to 0.000, which was less than 0.05. In addition, the impact of mindfulness training based on cognitive therapy was 95% (95% of variance was explained by the test implementation). Due to the undesirable impacts of different methods on weight reduction (regime, surgery, etc.), the psychological treatment was the most common and convenience method for obesity treatment [**28**]. Cognitive-behavioral approaches in the treatment have been considered as the center of gravity of medical researches on obesity in 1990’s [**29**]. Emotional eating was mentioned in explaining obesity in terms of psychology. In addition, it was said that overeating was a type of reaction to stress and anxiety that a person used in order to avoid other obese individuals or individuals with overweight when he was influenced by stress or emotional states. Generally, the mindfulness method had a great impact on obese individuals because mindfulness training led to cognitive changes in the way of thinking and actions of patients and took advantage of the conditional strengthening principals. Therefore, the patient made an effort to reach the next step and see himself above and this tendency to continuous improvement led to better conditions gradually. The finding was in line with the results obtained by Mousavian N, Moradi A, Mirzaie J, Sheidfar F, Mahmoudi B, Taheri F (2010), Dalen J et al (2010), Singh NN, Lancioni GE, Singh AN et al (2008), Kristeller JL, Hallet CB (1999), Tapper K, Shaw C, Ilsley J, Hill AJ, Bond FW, Moore L (2008) [**3**,**30**-**33**]. 

Third hypothesis: mindfulness training associated with dietary regime leads to weight loss in obese individuals.

According to the findings of the present research, performing mindfulness training associated with dietary regime had a greater impact compared to performing the variables alone (dietary regime and mindfulness training). The significance level was equal to 0.000, which was less than 0.05, and indicated that obesity after performing mindfulness training and dietary regime had a significant difference compared to the variable in the pretest, and obesity among individuals in the experiment group significantly decreased compared to the control group. The effect size of the variable was equal to 0.966, which meant that about 97% of the variance of the posttest could be explained by the mindfulness training associated with receiving the dietary regime. Psychological and mindfulness methods were the methods that could be used in weight loss. We all know that less should be eaten in order to have a healthy life but this simple rule is not obeyed most of the time and statistics approves this. This is because of neglecting the most vital and significant factor in weight reduction which is nothing but the “mind”. For instance, when the mind and body cooperate in a business, mind is the senior manager and body is an employee who follows the orders of his superiors. Mindfulness increases self-awareness, thereby the person will be in line with the works he/ she does. Mindfulness focuses mind, body, and spirit on the weight loss components and helps people achieve weight loss. This leads to the enhancement of their awareness of the actual needs of their body and its chain effects result in the promotion of mind ability and inform them about what their body need for real instead of unconscious eating. Daily mindfulness training not only controls the tendency to overeating but also motivates people to follow a dietary regime and walk on the road to health.

Fourth hypothesis: effectiveness of mindfulness training associated with dietary regime on weight loss is more than effectiveness of mindfulness training alone.

According to the research findings, the effect size of group, which was trained in mindfulness (0.948) was less than the effect size of group which used a combination of mindfulness training and dietary regime (0.966). Therefore, it could be concluded that mindfulness training had less than a second impact when it was used alone until the training was combined with a dietary regime.

Fifth hypothesis: effectiveness of dietary regime on weight loss of individuals has durability after 2 months.

The research findings indicated that the dietary regime alone during 2 months after the end of dietary cannot guarantee weight loss of individuals and durability. Here, this question arose that what happens after the end of the dietary regime? If we asked the question, the answer would be: I cannot have a proper dietary regime, it is observed a few days, and I forget it after a while. One of the reasons could be the fact that these persons are someone who think they have no control on their life events including on how to feed themselves and someone else must control them from outside. In this case, the external locus of control is a nutritionist. They observe their regime until they are under supervision of a nutritionist, but they return to previous bad nutritional habits as soon as they complete the course. Therefore, these individuals significantly believe that they cannot control their behaviors. The clinical challenge is not convincing these people regarding the fact that they need permanent training and education but the important challenge is to motivate these people based on the fact that the successful management of their weight demands a continuous control of emotions and thoughts, especially for individuals who might show an established pattern of overeating in reaction to environmental motivations. The findings were in line with the ones of Mousavian N, Moradi A, Mirzaie J, Sheidfar F, Mahmoudi B, Taheri F (2010), Kristeller JL, Hallet CB (1999) [**3**,**32**].

Sixth hypothesis: effectiveness of mindfulness and dietary regime on weight loss of individuals has durability after 2 months

According to the present research, individuals who received both mindfulness training and appropriate dietary regime had no weight gain after two months. Therefore, this combined training program had a great impact and it was even more durable than mindfulness training. Previous researches also approved the finding because individuals who received both mindfulness training and dietary regime were ready to reduce weight in terms of mental and physical and this mutual readiness led to more durability. The research findings indicated that performing a dietary regime and also mindfulness training based on cognitive therapy, each of them individually, and a combination of both of them (mindfulness training and dietary regime) all had an impact on the weight reduction of obese individuals. In the field of mindfulness, concentration was mostly on the individuals’ mind and these trainings motivated them to reduce their weight psychologically. These trainings mostly emphasized the hazards of obesity and how to treat them. It should be mentioned in the scope of mindfulness that most of psychological, religious, and philosophical points of view emphasized the importance of consciousness in maintaining and enhancing the well-being [**34**]. The concept of mindfulness is one of the features of consciousness that is in relation to well-being and there have been many discussions in this regard. Mindfulness refers to one’s clear awareness of what is within and in interaction with the external environment [**34**]. In total, it could be expressed that since mindfulness training is one of the interventions based on behavioral-cognitive therapies and performing a dietary regime is of its solutions, one could be ready for to eat less than before. When these two (dietary regime and mindfulness training) are combined with each other and individuals are under control both physically and psychologically in order to reduce weight, better results are obtained. The results of the present research confirmed what was mentioned above.
